# COVID-19 Relief Receipt and U.S. Household Food Expenditures

**DOI:** 10.1016/j.focus.2024.100265

**Published:** 2024-07-25

**Authors:** Bridget Yeboah Bafowaa, Andrea M. Leschewski

**Affiliations:** 1 Department of Agricultural Economics & Rural Sociology, Auburn University, Auburn, Alabama; 2 Ness School of Management and Economics, South Dakota State University, Brookings, South Dakota

**Keywords:** Food at home, food away from home, expenditures, emergency relief, pandemic

## Abstract

**Introduction:**

COVID-19 relief payments and programs may have contributed to the shift in food expenditures from food away from home to food at home during the pandemic. This shift has public health implications given the differences in the nutritional quality between food at home and food away from home. The objective of this study is to examine the association between COVID-19 relief and household food at home and food away from home expenditure shares. Economic Impact Payments, Pandemic-Electronic Benefits Transfer, Supplemental Nutrition Assistance Program, and charitable food receipt are considered.

**Methods:**

Food expenditure and COVID-19 relief data for 265,443 households were obtained from Phase 3.1 (April 28 to July 5, 2021) of the Household Pulse Survey. Poisson pseudo-maximum likelihood estimators were employed in 2023 to analyze the association between COVID-19 relief and household food at home and food away from home expenditure shares.

**Results:**

Pandemic-Electronic Benefits Transfer receipt was associated with households allocating 3% less of food expenditures to food at home and 9% more to food away from home. Supplemental Nutrition Assistance Program and charitable food receipt were associated with spending 8% and 3% more of food expenditures on food at home and 22% and 9% less on food away from home, respectively. Recent Economic Impact Payment receipt was associated with reduced food at home and increased food away from home expenditure shares among households with low-income and/or a Black respondent.

**Conclusions:**

Study results indicate that COVID-19 relief contributed to changing food expenditure patterns during the pandemic. Of note, Pandemic-Electronic Benefits Transfer and Economic Impact Payment receipt were associated with spending a greater share of food dollars on food away from home. Nutritional implications of COVID-19 relief warrant further investigation and should be carefully considered in the design of future nutrition assistance emergency relief.

## HIGHLIGHTS

• COVID-19 relief contributed to changing food expenditure patterns during the pandemic.
• Supplemental Nutrition Assistance Program and charitable food recipients allocated a greater share of total food expenditures to food at home.
• Pandemic-Electronic Benefits Transfer and Economic Impact Payments recipients spent a greater share of total food expenditures on less healthy food away from home.

## INTRODUCTION

Household expenditures on food away from home (FAFH) exceeded food at home (FAH) expenditures for the first time in the U.S. in 2010.^1^ FAFH refers to prepared food from full- or limited-service restaurants, whereas FAH is defined as food for home consumption from grocery stores, convenience stores, supercenters, dollar stores, and warehouse clubs. In the year prior to the coronavirus disease 2019 (COVID-19) pandemic, 56.2% of U.S. food expenditures were spent on FAFH, and 43.8% were spent on FAH.^2^ After the start of the pandemic in early 2020, there was a sizeable shift in food expenditure patterns. The share of food expenditures allocated to FAFH decreased by 15.8%, whereas the share allocated to FAH increased by 13.95%.^2^ This shift was, in part, attributable to shelter-in-place orders, business closures (including restaurants), fear of contagion, increasing unemployment, and declining income.^3^^,^^4^

Food expenditure patterns were likely further influenced by COVID-19 relief payments and programs, which provided benefits specifically for food purchase as well as cash payments that increased income available to purchase food. The Coronavirus Aid, Relief, and Economic Security Act; the COVID-related Tax Relief Act of 2020; and the American Rescue Plan of 2021 authorized a series of 3, 1-time Economic Impact Payments (EIP) that assisted eligible individuals and families with income loss and expenses caused by the pandemic.^5^ Recent studies find that approximately 65% of recipients allocated some of their EIP to purchase food, with an estimated 33% of the total EIP spent on food.^6^^,^^7^ The American Rescue Plan further increased the Child Tax Credit (CTC) and expanded its scope to reduce child poverty by supplementing household income. The National Bureau of Economic Research found that CTC expansion increased food spending, with an estimated 28% of CTC payments spent on food.^8^

Existing food assistance programs were also expanded and modified during the pandemic. The Supplemental Nutrition Assistance Program (SNAP) provides benefits to low-income families on Electronic Benefits Transfer (EBT) cards, which can be used to purchase food at SNAP-authorized retailers. During the pandemic, Congress temporarily raised SNAP benefits by 15% and increased all households to the maximum benefit level for their respective household size; households at or close to the maximum benefit level prior to the pandemic received at least a $95 benefit increase.^9^ Congress also created a new program, Pandemic EBT (P-EBT), which provided benefits to families whose children lost access to meals provided by school meal programs. P-EBT benefits were equivalent to the value of school meals missed and were distributed on EBT cards that could be used to purchase food at SNAP-authorized retailers.^10^ Complementing government assistance, household charitable food receipt from food banks and other charitable organizations also increased during the pandemic.^11^^,^^12^ Estimates indicate a 26% increase in the pounds of food distributed by food banks between March 2019 and March 2020.^12^

Understanding how COVID-19 relief contributed to the pandemic-induced food expenditure shift is of particular interest given differences in the nutritional quality of FAFH and FAH. FAFH tends to contain more calories and nutrients of public health concern than FAH.^13^ Consuming FAFH is also associated with greater odds of obesity and higher cardiovascular disease biomarkers.^14^ Given the nutritional focus of SNAP and school meal programs, P-EBT and SNAP were designed to be used only for FAH purchases. However, P-EBT and SNAP receipt may have resulted in increased FAFH spending by freeing other household income for the purchase of FAFH. In contrast, EIP were cash payments that recipients could use to purchase any good or service, including both FAH and FAFH. With approximately 65% of recipients making food purchases with EIP, it is important to consider the public health implications of EIP receipt resulting from household allocation of EIP payments to FAH and FAFH.^7^

The literature on COVID-19 relief and food expenditures is limited. Analyses of EIP indicate that its receipt is associated with increased food expenditures but that this relationship varies across time.^15^^,^^16^ Receipt of the first and second EIP was associated with increased FAH spending, but only the first EIP was associated with increased FAFH spending.^16^ The third EIP payment was not significantly (*p<*0.10) associated with either type of food expenditures.^16^ The impact of SNAP participation and benefit expansion on food expenditures has been examined using prepandemic data. Results indicate that SNAP participation is associated with increased FAH and decreased FAFH expenditures.^17^^,^^18^ Further SNAP benefit expansion is associated with an increase in both FAH and FAFH expenditures.^19^ P-EBT, SNAP, and charitable food receipt's effect on food expenditures have not been examined during the pandemic period.

The objective of this study is to examine the association between COVID-19 relief and the share of household food expenditures allocated to FAH and FAFH. EIP, P-EBT, SNAP, and charitable food receipt are considered. Understanding the association between COVID-19 relief and FAH and FAFH expenditure shares is needed to inform the design of future emergency food relief that effectively supports the purchase of nutritious food.

## METHODS

### Study Sample

This study uses publicly available, deidentified data from the Household Pulse Survey (HPS). The HPS is a nationally representative survey designed and administered by the U.S. Census Bureau to measure the impact of COVID-19 on household social and economic conditions. HPS data collection began in April 2020 and currently consists of 15 phases. This study utilizes data from Phase 3.1, which includes 12 weeks of data collected between April 28, 2021 and July 5, 2021. Phase 3.1 is the only HPS phase to simultaneously measure household FAH and FAFH expenditures, EIP, SNAP, P-EBT, and charitable food receipt. The final study sample consisted of 265,443 households.

### Measures

The main measures of interest in this analysis were household FAH and FAFH expenditure shares, that is, household weekly FAH and FAFH expenditures as a share of total weekly food expenditures. HPS respondents were asked to indicate in the past 7 days how much they and their household spent on food at supermarkets, grocery stores, online, and other places they buy food to prepare and eat at home (i.e., FAH) and how much they spent on prepared meals, fast food, and carry out or delivered meals (i.e., FAFH). Total food expenditures were calculated by summing household FAH and FAFH expenditures. FAH and FAFH expenditure shares were then calculated by dividing both FAH and FAFH expenditures by total food expenditures.

The HPS also measured the receipt of the COVID-19 relief considered in this analysis: EIP, P-EBT, SNAP, and charitable food. Note that CTC receipt was not considered because it was not distributed during the time period analyzed in this study. All relief measures were binary indicators equaling 1 if a household received the relief and 0 otherwise. In the HPS, households indicated whether they currently receive SNAP or P-EBT and whether they received EIP or charitable food in the past 7 days. Charitable food was defined in the HPS as free groceries from a food pantry, food bank, church, or other place that helps with free food.

Other covariates included demographics and state and week indicators. Demographics considered were the respondent's age, sex, race/ethnicity, marital status, and education as well as household income and the number of adults and children in the household.

### Statistical Analysis

The Poisson pseudo-maximum likelihood (PPML) estimator was used to analyze the association between COVID-19 relief package receipt and household FAH and FAFH expenditure shares. The PPML estimator was chosen because it accommodates corner solutions, which in the context of this analysis corresponded to households who allocated 0% of their food expenditures to FAH and/or FAFH. In total, 16% of the study sample allocated 0% of their food expenditures to FAFH, and 2% allocated 0% of their expenditures to FAH. Two primary models were estimated through PPML, with FAH expenditure share and FAFH expenditure share serving as the dependent variable in each respective model. COVID-19 relief, household demographics, and state and week indicators were included as covariates in both models. Subsample analyses were further conducted to examine potential differences in food expenditure responses to COVID-19 relief receipt across race, ethnicity, and income. Household-level survey weights were used in all analyses to obtain nationally representative estimates.

After estimation, the HPC test was applied to compare the appropriateness of the PPML estimator with an alternative corner solution model, Heckman's sample selection estimator.^20^ The Heckman model results were consistent with PPML estimates. HPC test results strongly rejected the Heckman estimator and indicated that the PPML estimator was preferred. Linear and tobit regressions were also run as an additional robustness check; resulting estimates were consistent with those obtained using PPML. All analyses were conducted in 2023 in Stata, Version 18.0.

## RESULTS

Weighted descriptive statistics are provided in Table 1. Data from HPS Phase 3.1 provide a sample of 265,443 households. These exclude observations with incomplete or missing data. On average, households spent 72% of their food expenditures on FAH and 28% on FAFH in the past 7 days.

**Table 1:** Weighted Descriptive Statistics of Sample Households

Variable	Observations	Mean/proportion^a^	SD	Minimum	Maximum
Food expenditure shares					
FAH expenditure share	265,443	0.72	0.21	0	1
FAFH expenditure share	265,443	0.28	0.21	0	1
Relief packages					
SNAP	265,443	0.11	—	0	1
Charitable food	265,443	0.07	—	0	1
P-EBT	265,443	0.02	—	0	1
EIP	265,443	0.14	—	0	1
Household characteristics					
Age (years)	265,443	51.88	—	18	88
Male	265,443	0.47	—	0	1
Education	265,443	0.46	—	0	1
Marital status	265,443	0.52	—	0	1
Number of kids	265,443	0.51	1.00	0	5
Number of adults	265,443	2.10	0.95	1	10
Black	265,443	0.11	—	0	1
White	265,443	0.80	—	0	1
Other	265,443	0.09	—	0	1
Hispanic	265,443	0.13	—	0	1
Income ($0–$100,000)	265,443	0.70	—	0	1
Income ($100,000–$199,000)	265,443	0.22	—	0	1
Income ≥$200,000	265,443	0.08	—	0	1

a Mean and SD are provided for continuous variables, and proportions are provided for discrete variables. Household-level sampling weights were employed. EIP, Economic Impact Payment; FAH, food at home; FAFH, food away from home; P-EBT, Pandemic Electronic Benefits Transfer; SNAP, Supplemental Nutrition Assistance Program.

Of the sample households, 14% indicated that they received an EIP payment, and 7% received charitable food in the past 7 days. In addition, 11% of sample households indicated that at least 1 household member received SNAP benefits, and 2% received P-EBT. The relatively low share of households receiving P-EBT likely reflects that more than half of states first P-EBT disbursement occurred after May 2021 for the 2020–2021 academic year.^21^ The average survey respondent was female (53%), White (80%), non-Hispanic (87%), and married (52%). The average sample household consisted of 2 adults and had an annual income below $100,000 (70%).

Tables 2 and 3 provide PPML estimates of the association between COVID-19 relief and household FAH and FAFH expenditure shares, respectively. Estimated coefficients are presented in Column 1 of each table, whereas Column 2 presents transformed coefficients representing the expected percentage change for interpretation. The PPML estimates indicate that COVID-19 relief receipt was significantly associated with both FAH and FAFH expenditure shares. Shown in Table 2, SNAP and charitable food receipts were associated with 8% and 3% higher FAH expenditures shares, respectively. That is, respondents whose household participated in SNAP or received charitable food in the past 7 days allocated a greater share of their total food expenditures to FAH. In contrast, P-EBT receipt was associated with households allocating 3% less of their total food expenditure to FAH.

**Table 2:** PPML Estimates (Dependent Variable = FAH Share)

Measure	(1) Coefficient (95% CI)^a^	(2) Percentage change (95% CI)^b^
SNAP	**0.08 (0.07, 0.09)**	**0.08 (0.07, 0.09)**
EIP	0.00 (−0.01, 0.01)	0.00 (−0.01, 0.01)
P-EBT	−**0.03 (**−**0.04,** −**0.01)**	−**0.03 (**−**0.04,** −**0.01)**
Charitable food	**0.03 (0.02, 0.04)**	**0.03 (0.02, 0.04)**
Constant	−**0.43 (**−**0.45,** −**0.41)**	−**0.35 (**−**0.36,** −**0.34)**
Wald chi-square	5,155.72	
Prob > chi-square	0.00	
Observations	265,443	

*Note:* Boldface indicates significance at the 10% level.

a Household characteristics and state and week indicators were included as controls. Household-level sampling weights were employed. Complete results can be found in Appendix Table 1 (available online).

b Percentage change = exp(coefficient)−1. EIP, Economic Impact Payments; FAH, food at home; P-EBT, Pandemic Electronic Benefits Transfer; PPML, Poisson pseudo-maximum likelihood; Prob, probability; SNAP, Supplemental Nutrition Assistance Program.

**Table 3:** PPML (Dependent Variable = FAFH Share)

Measure	(1) Coefficient (95% CI)^a^	(2) Percentage change (95% CI)^b^
SNAP	**−0.25 (−0.28, −0.22)**	**−0.22 (−0.24, −0.20)**
EIP	0.00 (−0.01, 0.02)	0.00 (−0.01, 0.02)
P-EBT	**0.09 (0.04, 0.13)**	**0.09 (0.04, 0.14)**
Charitable food	−**0.09 (**−**0.12,** −**0.06)**	−**0.09 (**−**0.11,** −**0.06)**
Constant	−**1.02 (**−**1.07,** −**0.98)**	−**0.64 (**−**0.66,** −**0.63)**
Wald chi-square	5,048.21	
Prob > chi-square	0.00	
Observations	265,443	

*Note:* Boldface indicates significance at the 10% level.

a Household characteristics and state and week indicators were included as controls. Household-level sampling weights were employed. Complete results can be found in Appendix Table 2 (available online).

b Percentage change = exp(coefficient)−1. EIP, Economic Impact Payments; FAFH, food away from home; P-EBT, Pandemic Electronic Benefits Transfer; PPML, Poisson pseudo-maximum likelihood; Prob, probability; SNAP, Supplemental Nutrition Assistance Program.

Similarly, results detailed in Table 3 suggest that SNAP and charitable food receipts were associated with FAFH expenditure shares. Receipt of SNAP was associated with a 22% decrease in FAFH expenditures shares, whereas charitable food receipt in the past 7 days was associated with a 9% decrease. In contrast, P-EBT receipt was associated with a 9% increase in household FAFH expenditure shares. Results indicate that EIP receipt in the past 7 days was not significantly (*p*<0.10) associated with household FAH or FAFH expenditure shares.

Subsample analyses examining potential differences in food expenditure responses to COVID-19 relief receipt across race, ethnicity, and income are presented in Table 4, Table 5, Table 6. Tables 4 and 5 provide PPML estimates of the association between COVID-19 relief and household FAH and FAFH expenditure shares by race and ethnicity. Results indicate that SNAP and charitable food receipts are associated with increased allocation of food expenditures to FAH and decreased allocation to FAFH for all races and ethnicities.

**Table 4:** PPML Subsample Analysis—Race and Ethnicity (Dependent Variable = FAH Share)

	Race			Ethnicity	
Measure	White % change (95% CI)^a^^,^^b^	Black % change (95% CI)^a^^,^^b^	Other % change (95% CI)^a^^,^^b^	Hispanic % change (95% CI)^a^^,^^b^	Non-Hispanic % change (95% CI)^a^^,^^b^
SNAP	**0.08 (0.07, 0.09)**	**0.11 (0.08, 0.13)**	**0.06 (0.03, 0.08)**	**0.05 (0.03, 0.07)**	**0.09 (0.08,0.09)**
EIP	0.01 (0.00, 0.01)	−**0.02 (**−**0.04, 0.00)**	−0.01 (−0.03, 0.01)	−0.01 (−0.02, 0.01)	0.00 (−0.01, 0.01)
P-EBT	−**0.02 (**−**0.04, 0.00)**	−**0.03 (**−**0.07, 0.01)**	−0.03 (−0.06, 0.01)	−0.02 (−0.05, 0.01)	−**0.03 (**−**0.05,** −**0.01)**
Charitable food	**0.03 (0.02, 0.04)**	**0.02 (0.00,** −**0.04)**	**0.02 (**−**0.00, 0.05)**	**0.04 (0.02, 0.06)**	**0.03 (0.02, 0.04)**
Constant	−**0.35 (**−**0.36,** −**0.34)**	−**0.41 (**−**0.44,** −**0.38)**	−**0.34 (**−**0.39,** −**0.30)**	−**0.34 (**−**0.41,** −**0.28)**	−**0.35 (**−**0.36,** −**0.34)**
Wald chi-square	4,707.25	488.42	480.95	422.17	4,972.00
Prob > chi-square	0.00	0.00	0.00	0.00	0.00
Observations	222,787	18,138	24,518	21,212	244,231

*Note:* Boldface indicates significance at the 10% level.

a Household characteristics and state and week indicators were included as controls. Household-level sampling weights were employed.

b Percentage change = exp(coefficient)−1. EIP, Economic Impact Payments; FAH, food at home; P-EBT, Pandemic Electronic Benefits Transfer; PPML, Poisson pseudo-maximum likelihood; Prob, probability; SNAP, Supplemental Nutrition Assistance Program.

**Table 5:** PPML Subsample Analysis—Race and Ethnicity (Dependent Variable = FAFH Share)

	Race			Ethnicity	
Measure	White % change (95% CI)^a^^,^^b^	Black % change (95% CI)^a^^,^^b^	Other % change (95% CI)^a^^,^^b^	Hispanic % change (95% CI)^a^^,^^b^	Non-Hispanic % change (95% CI)^a^^,^^b^
SNAP	**−0.23 (**−**0.26,** −**0.21)**	−**0.25 (**−**0.29,** −**0.21)**	−**0.15 (**−**0.21,** −**0.09)**	−**0.13 (**−**0.19,** −**0.07)**	−**0.24 (**−**0.27,** −**0.22)**
EIP	−0.02 (−0.04, 0.01)	**0.06 (0.01, 0.11)**	0.02 (−0.02, 0.07)	0.01 (−0.04, 0.06)	0.00 (−0.01, 0.02)
P-EBT	**0.07 (0.01, 0.13)**	**0.09 (0.00, 0.21)**	0.07 (−0.03, 0.17)	0.05 (−0.04, 0.14)	**0.11 (0.05, 0.16)**
Charitable food	−**0.10 (**−**0.13,** −**0.07)**	−**0.07 (**−**0.12, 0.00)**	−**0.06 (**−**0.12, 0.00)**	−**0.10 (**−**0.16,** −**0.04)**	−**0.09 (**−**0.11,** −**0.05)**
Constant	−**0.64 (**−**0.66,** −**0.63)**	−**0.55 (**−**0.61,** −**0.48)**	−**0.65 (**−**0.71,** −**0.58)**	−**0.65 (**−**0.73,** −**0.56)**	−**0.64 (**−**0.65,** −**0.62)**
Wald chi-square	4,589.82	413.64	473.66	437.64	4,757.14
Prob > chi-square	0.00	0.00	0.00	0.00	0.00
Observations	222,787	18,138	24,518	21,212	244,231

*Note:* Boldface indicates significance at the 10% level.

a Household characteristics and state and week indicators were included as controls. Household-level sampling weights were employed.

b Percentage change = exp(coefficient)−1. EIP, Economic Impact Payments; FAFH, food away from home; P-EBT, Pandemic Electronic Benefits Transfer; PPML, Poisson pseudo-maximum likelihood; Prob, probability; SNAP, Supplemental Nutrition Assistance Program.

**Table 6:** PPML Subsample Analysis—Incomes (Dependent Variables = FAH Expenditure Share and FAFH Expenditure Share)

	FAH		FAFH	
Measure	< 200% FPL % change (95% CI)^a^^,^^b^	≥ 200% FPL % change (95% CI)^a^^,^^b^	< 200% FPL % change (95% CI)^a^^,^^b^	≥ 200% FPL % change (95% CI)^a^^,^^b^
SNAP	**0.08 (0.07, 0.09)**	**—**	**−0.23 (−0.26, −0.20)**	**—**
EIP	**−0.02 (−0.03, 0.00)**	0.00 (0.00, 0.01)	**0.06 (0.01, 0.10)**	−0.01 (−0.03, 0.01)
P-EBT	**−0.02 (−0.04, 0.00)**	0.00 (**−**0.01, 0.03)	**0.08 (0.01, 0.15)**	−0.01 (−0.07, 005)
Charitable food	**0.01 (0.00, 0.03)**	**0.04 (0.03, 0.06)**	**−0.04 (−0.09, −0.01)**	**−0.11 (−0.14, −0.09)**
Constant	**−0.32 (−0.35, −0.30)**	**−0.37 (−0.38, −0.36)**	**−0.67 (−0.71, −0.62)**	**−0.62 (−0.64, −0.60)**
Wald chi-square	898.66	3,530.24	916.30	3,656.20
Prob > chi-square	0.00	0.00	0.00	0.00
Observations	38,198	227,245	38,198	227,245

*Note:* Boldface indicates significance at the 10% level.

a Household characteristics and state and week indicators were included as controls. Household-level sampling weights were employed.

b Percentage change = exp(coefficient)−1. EIP, Economic Impact Payments; FAH, food at home; FAFH, food away from home; FPL, Federal Poverty Line; P-EBT, Pandemic Electronic Benefits Transfer; PPML, Poisson pseudo-maximum likelihood; Prob, probability; SNAP, Supplemental Nutrition Assistance Program.

However, receipt of EIP is only significantly (*p*<0.10) associated with FAH and FAFH expenditures shares for households with a Black respondent; for these households, EIP receipt is associated with a 2% decrease in FAH expenditure shares and a 6% increase in FAFH expenditure shares. P-EBT receipt is associated with decreased FAH expenditure shares and increased FAFH expenditure shares for households with a White, Black, and/or non-Hispanic respondents. In contrast, results indicate no significant association between P-EBT receipt and food expenditure shares among households with respondents who identified as Hispanic or other race.

Table 6 provides PPML estimates of the association between COVID-19 relief and household FAH and FAFH expenditure shares by household income. Households with income below 200% of the Federal Poverty Line (FPL) are compared with households with income at or above 200% FPL. Results suggest that the association between COVID-19 relief receipt and food expenditure shares varies with income for some forms of relief. EIP and P-EBT receipt are associated with a reduced FAH expenditure shares and increased FAFH expenditure shares among households with income below 200% FPL. In contrast, EIP and P-EBT receipt are not significantly (*p*<0.10) associated with food expenditure shares among households with income at or above 200% FPL. The association between charitable food receipt and FAH and FAFH expenditure shares is consistent across both income groups. SNAP receipt is associated with an 8% increase in FAH expenditure shares and 23% decrease in FAFH expenditure shares among households with income below 200% FPL. Note that SNAP is not included as a covariate in the PPML model for households with income at or greater than 200% FPL owing to program eligibility requirements.

## DISCUSSION

This study analyzed the association between COVID-19 relief and U.S. household FAH and FAFH expenditure shares. The effect of EIP, P-EBT, SNAP, and charitable food receipt was considered. Results indicate that SNAP and charitable food receipts were associated with households allocating a greater share of food expenditures to FAH and a lower share of expenditures to FAFH. In contrast, households receiving P-EBT allocated a greater share of food dollars to FAFH and a lower share to FAH. Receipt of EIP in the past 7 days was associated with increased FAFH and decreased FAH expenditure shares among households with income below 200% FPL and/or with a Black respondent.

P-EBT was a key component of the food safety net during the pandemic, with receipt decreasing food insufficiency among SNAP households by 28% and very low food security among children by 17%.^21^ However, results from this study indicate that the nutritional implications of P-EBT receipt should be considered. P-EBT was designed for FAH purchases, with the program providing families with the value of missed school meals on EBT cards to be used at SNAP-authorized food retailers. Yet, results indicate that P-EBT had an unintended, positive association with FAFH expenditure shares, particularly among households with income below 200% FPL and those with a White, Black, and/or non-Hispanic respondent. That is, P-EBT receipt was associated with a 2% decrease in household FAH expenditure shares and a 6% increase in FAFH expenditure shares. This finding likely reflects that P-EBT receipt freed household income previously used to purchase FAH, allowing households to increase FAFH expenditures.

The positive association between P-EBT receipt and household FAFH expenditure shares indicates that the program may have had a negative impact on nutritional quality. Breakfasts and lunches provided by the National School Lunch Program and School Breakfast Program are required to meet established meal pattern requirements on the basis of the *Dietary Guidelines for Americans*. FAFH, in contrast, is associated with increased intake of energy and nutrients that pose harm to health, including saturated fat, total fat, sugars, and sodium.^13^ Thus, by inducing increased FAFH expenditure shares, P-EBT may have had the unintentional effect of reducing the nutritional quality of food consumed by children during the pandemic.

Households receiving P-EBT may have increased the share of food expenditures allocated to FAFH given the unique challenges they faced due to school closures during the pandemic.^22^^,^^23^ Many parents were tasked with balancing working from home and child care.^22^ FAFH may have been more feasible for these households because it was likely challenging for parents in this situation to prepare breakfasts and lunches, previously supplied by schools, while also teleworking and managing their children's remote learning. Increased FAFH expenditure shares might also be associated with the increased share of school-aged children left at home unsupervised during the pandemic.^24^^,^^25^ Parents with limited job flexibility and child care options often had to resort to leaving their children unsupervised at home.^24^^,^^25^ In this situation, parents may have increased FAFH expenditure shares because preparing home cooked meals would have been challenging for younger children.

Future research should focus on analyzing the mechanism linking P-EBT receipt to FAFH and FAH expenditure shares as well as corresponding changes to diet quality. The impact of alternative school meal relief delivery modes (e.g., pick-up and delivery) should also be considered. Collectively, this research will inform future school meal program emergency relief policy by providing insight on which delivery modes support the National School Lunch Program's and School Breakfast Program's goals of providing nutritionally balanced, low-cost, or free lunches and breakfasts to children on each school day.

In contrast with P-EBT, SNAP receipt was associated with increased FAH expenditure shares and decreased FAFH expenditure shares. This finding is consistent with the prepandemic literature on SNAP's effect on food expenditure allocation.^17^, ^18^, ^19^ Results indicate that SNAP receipt had the intended effect of increasing FAH spending and that food expenditure shifts induced by SNAP align with the program's goal of helping families with low income afford and access nutritious food.

Study results also provide initial evidence that charitable food receipt from food pantries and other organizations in the past week was associated with increased FAH expenditure shares and decreased FAFH expenditure shares. Increased FAH expenditure shares may reflect that households complement food items obtained from food pantries with additional FAH items to prepare a complete meal(s). This result suggests that charitable food may serve as an effective means to simultaneously distribute emergency food aid and improve diet quality by inducing recipients to shift food expenditures from less healthy FAFH to FAH. Additional research is needed to understand the factors driving the relationship between charitable food receipt and food expenditure shares and to provide insight on charitable food system infrastructure and resource investment as a potential policy response to future food system disruptions and national emergencies.^26^

Findings further suggest that EIP receipt in the past week was associated with household food expenditure allocation. This study considered relief received between April 28 and July 5, 2021, which coincided with distribution of the third EIP. EIP receipt was associated with decreased FAH expenditure shares and increased FAFH expenditure shares among households with income below 200% FPL and/or with a Black respondent. This result contrasts with Bina et al.^16^ (2023)’s finding that the third EIP had no significant effect on FAH or FAFH expenditures. The relationship between EIP receipt and food expenditure allocation across race and income warrants further consideration given that FAFH tends to have lower nutritional quality than FAH and the higher incidence of diet-related chronic diseases and conditions among minority and low-income populations.^27^ EIP modifications to encourage FAH expenditures should also be examined. Given that 33% of EIP payments are redeemed on food, policymakers and researchers responding to future national emergencies might consider distributing a share of the EIP through SNAP.^7^

### Limitations

Study results should be interpreted subject to the following limitations. All analysis was conducted using cross-sectional data, and results thus indicate association, not causality, between COVID-19 relief receipt and food expenditure shares. Data constraints also posed a challenge. Question variation across HPS phases limited the focus of this study to a 3-month period of the pandemic, during which some forms of COVID-19 relief, including CTCs, were not distributed. The HPS data were further limited because they measured EIP receipt in the past 7 days despite this form of relief being distributed on an infrequent basis. Results for EIP should thus be interpreted as the association between food expenditure shares and recent EIP receipt.

Food expenditure and SNAP participation measurement error in the HPS is also a concern. The literature indicates that SNAP participation and consumption expenditures are often underreported in large, nationally representative data sets similar to the HPS.^28^^,^^29^ Both forms of underreporting would likely reduce the magnitude of the association between COVID-19 relief receipt and food expenditure shares. It is also important to note that the HPS does not provide measures of nutritional quality for FAFH and FAH purchases. References to the nutritional quality of FAFH relative to that of FAH in the manuscript are based on findings from the literature and not the HPS data.^13^

## CONCLUSIONS

This study provides evidence that COVID-19 relief was associated with the shift in food expenditures allocated to FAH and FAFH during the pandemic. SNAP and charitable food receipts were associated with increased household FAH and decreased FAFH expenditure shares. P-EBT receipt, in contrast, was associated with spending a smaller share of food expenditures on FAH and a greater share on FAFH. EIP receipt was similarly associated with increased FAFH expenditure shares and decreased FAH expenditure shares among households with income below 200% FPL and/or with a Black respondent. P-EBT and EIP receipts’ associations with food expenditure allocation are noteworthy given that the literature indicates that FAFH tends to have lower nutritional quality than FAH.^13^ Nutritional implications of COVID-19 relief warrant further investigation and should be carefully considered in the design of future nutrition assistance emergency relief.

## Appendix Supplementary materials

Image, application 1

## Footnotes

Supplementary material associated with this article can be found in the online version at doi:10.1016/j.focus.2024.100265.
