# Supplementary material for: COVID-19 Relief Receipt and U.S. Household Food Expenditures
Source: AJPM Focus. 2024 Jul 25;3(5):100265. doi: 10.1016/j.focus.2024.100265 (PMC11388698; doi:10.1016/j.focus.2024.100265)
Supplement: Supplementary file 1 [file mmc1.docx]

**APPENDIX**

**Appendix Table 1**: Complete Poisson Pseudo-Maximum Likelihood Estimates; Dependent Variable = FAH Share

|  |  | (1)  Coefficient (95% CI)^a^ | (2)  Percentage Change  (95% CI)^b^ | |
| --- | --- | --- | --- | --- |
| SNAP |  | **0.08 (0.07, 0.09)** | **0.08 (0.07, 0.09)** | |
| EIP |  | 0.00 (-0.01, 0.01) | 0.00 (-0.01, 0.01) | |
| P-EBT |  | **-0.03 (-0.04, -0.01)** | **-0.03 (-0.04, -0.01)** | |
| Charitable Food |  | **0.03 (0.02, 0.04)** | **0.03 (0.02, 0.04)** | |
| Age (Years) |  | **0.00 (0.00, 0.00)** | **0.00 (0.00,0.00)** | |
| Male |  | **-0.03 (-0.03, -0.02)** | **-0.03 (-0.03, -0.02)** | |
| Education |  | **-0.02 (-0.02, -0.02)** | **-0.02 (-0.02, -0.02)** | |
| Marital Status |  | 0.00 (-0.00, 0.01) | 0.00 (-0.00, 0.01) | |
| Number of Kids |  | **0.02 (0.01, 0.02)** | **0.02 (0.01, 0.02)** | |
| Number of Adults |  | -0.00 (-0.00, 0.00) | -0.00 (-0.00, 0.00) | |
| Black |  | **-0.04 (-0.05, -0.03)** | **-0.04 (-0.05, -0.03)** | |
| Other |  | **-0.02 (-0.03, -0.01)** | **-0.02 (-0.03, -0.01)** | |
| Hispanic |  | **-0.03 (-0.04, -0.02)** | **-0.03 (-0.04, -0.02)** | |
| Income ($100,000- $199,000) |  | **-0.06 (-0.06, -0.05)** | **-0.05 (-0.06, -0.05)** | |
| Income $200,000+ |  | **-0.11 (-0.11, -0.10)** | **-0.10 (-0.11, -0.10)** | |
| Constant |  | **-0.43 (-0.45, -0.41)** | **-0.33 (-0.36, -0.34)** | |
| Wald Chi2 |  | 5,155.72 |  |  |
| Prob > Chi2 |  | 0.00 |  |  |
| Observations |  | 265,443 |  |  |

Boldface indicates significance at the 10% level.

*FAH, food at home;* CI, confidence interval; *SNAP, Supplemental Nutrition Assistance Program; EIP, Economic Impact Payments; P-EBT, Pandemic Electronic Benefits Transfer.*

*^a^State and week indicators were included as controls. Household-level sampling weights were employed.*

*^b^Percentage change = exp(coefficient)-1*

**Appendix Table 2**: Complete Poisson Pseudo-Maximum Likelihood; Dependent Variable = FAFH Share

|  |  | (1)  Coefficient (95% CI)^a^ | (2)  Percentage Change  (95% CI)^b^ | |
| --- | --- | --- | --- | --- |
| SNAP |  | **-0.25 (-0.28, -0.22)** | **-0.22 (-0.24, -0.20)** | |
| Economic Impact Payment |  | 0.00 (-0.01, 0.02) | 0.00 (-0.01, 0.02) | |
| P-EBT |  | **0.09 (0.04, 0.13)** | **0.09 (0.04, 0.14)** | |
| Charitable Food |  | **-0.09 (-0.12, -0.06)** | **-0.09 (-0.11, -0.06)** | |
| Age (Years) |  | **-0.01 (-0.01, -0.01)** | **-0.01 (-0.01, -0.01)** | |
| Male |  | **0.07 (0.05, 0.08)** | **0.07 (0.05, 0.08)** | |
| Education |  | **0.05 (0.04, 0.06)** | **0.05 (0.04, 0.06)** | |
| Marital Status |  | -0.00 (-0.01, 0.01) | -0.00 (-0.01, 0.01) | |
| Number of Kids |  | **-0.04 (-0.05, -0.04)** | **-0.04 (-0.05, -0.04)** | |
| Number of Adults |  | -0.00 (-0.01, 0.01) | -0.00 (-0.01, 0.01) | |
| Black |  | **0.10 (0.08, 0.12)** | **0.11 (0.08, 0.13)** | |
| Other |  | **0.04 (0.02, 0.06)** | **0.04 (0.02, 0.06)** | |
| Hispanic |  | **0.07 (0.05, 0.09)** | **0.07 (0.05, 0.09)** | |
| Income ($100,000- $199,000) |  | **0.14 (0.12, 0.15)** | **0.15 (0.13, 0.16)** | |
| Income $200,000+ |  | **0.24 (0.23, 0.26)** | **0.28 (0.26, 0.30)** | |
| Constant |  | **-1.02 (-1.07, -0.98)** | **-0.64 (-0.66, -0.63)** | |
| Wald Chi2 |  | 5,048.21 |  |  |
| Prob > Chi2 |  | 0.00 |  |  |
| Observations |  | 265,443 |  |  |

Boldface indicates significance at the 10% level.

*FAFH, food away from home;* CI, confidence interval; *SNAP, Supplemental Nutrition Assistance Program; EIP, Economic Impact Payments; P-EBT, Pandemic Electronic Benefits Transfer.*

*^a^State and week indicators were included as controls. Household-level sampling weights were employed.*

*^b^Percentage change = exp(coefficient)-1*
